# External Evaluation of Population Pharmacokinetic Models of Cabotegravir, During Its Oral and Intramuscular Administration in HIV‐Infected Patients

**DOI:** 10.1002/psp4.70180

**Published:** 2026-05-22

**Authors:** Quentin Renou, Nadège Néant, Alexandre Destere, Jennifer Lagoutte‐Renosi, Matthieu Grégoire, François Parant, Sébastien Lalanne, Florian Lemaitre, Peggy Gandia, Nicolas Venisse, Stéphane Bouchet, Minh P. Lê, Patrice Muret, Gilles Peytavin, Caroline Solas, Sihem Benaboud, the CARLA Study Group, Benoît Bailly, Vincent Gendrin, Timothhee Klopenstein, Souheil Zayet, Quentin Lepiller, Fabienne Bozon, Anne‐Sophie Brunel, Bruno Hoen, Laurent Hustache‐Mathieu, Camille Tumiotto, Mojgane Hemssamfar, Fabrice Bonnet, Ali Si‐Mohammed, Lionel Piroth, Marielle Buisson, Sophie Mahy, Michel Duong, Vinca Icard, Mary‐Anne Trabaud, Dulce Alfaiate, Agathe Becker, Evelyne Braun, Florence Brunel, Matthieu Godinot, Thomas Perpoint, Clément Javaux, Céline Boschi, Philippe Colson, Anne Motte, Véronique Obry‐Roguet, Sylvie Brégigeon, Olivia Faucher‐Zaegel, Hélène Laroche, Maeva Dos Santos, Isabelle Ravaux, Catherine Dhiver, Christelle Tomei, Amélie Ménard, Matthieu Million, Patrick Philibert, Christina Psomas, Jakub Kowalczyk, Simona‐Loredana Berbescu, Catherine Michel, Elisabeth André‐Garnier, Thomas Drumel, Clotilde Allavena, Eric Billaud, Sabelline Bouchez, Cécile Brunet‐Cartier, Colin Deschanvres, François Raffi, Véronique Reliquet, Gwennaelle Querne, Cecile Mear‐Passard, Antoine Asquier, Charlotte Moyon, Virginie Beslon, Lio Collias, Anne‐Geneviève Marcelin, Vincent Calvez, Diane Descamps, Valentine Ferré, Yazdanpanah Yazdan, Jade Ghosn, Valérie Pourcher, Marc‐Antoine Valantin, Gwenaël Le Moal, David Plainchamp, Anne Maillard, François Benezit, Cédric Arvieux, Pierre Gantner, Christine Cheneau, Axel Ursenbach, Baptiste Hoellinger, Céline Melounou, David Rey, Yves Hansmann

**Affiliations:** ^1^ Unité des Virus Émergents (UVE: Aix‐Marseille Univ‐IRD 190‐Inserm 1207) Marseille France; ^2^ APHM, Hôpital La Timone, Laboratoire de Pharmacocinétique et Toxicologie, Unité des Virus Émergents (UVE: Aix‐Marseille Univ‐IRD 190‐Inserm 1207) Marseille France; ^3^ Department of Clinical Pharmacology Université Côte d'Azur Medical Centre Nice France; ^4^ Université Côte d'Azur, Inria, CNRS, Laboratoire J.A. Dieudonné, Maasai Team Nice France; ^5^ Université Marie et Louis Pasteur, CHU Besançon, SINERGIES (UR 4662) Besançon France; ^6^ Nantes Université, CHU Nantes, Service de Pharmacologie Clinique Nantes France; ^7^ Nantes Université, CHU Nantes, Cibles et médicaments des Infections et de l'immunité, 9 IICiMed, UR 1155 Nantes France; ^8^ Hospices Civils de Lyon, Groupement Hospitalier Sud, Service de Biochimie et Biologie Moléculaire, UM Pharmacologie‐Toxicologie Pierre‐Benite France; ^9^ Univ Rennes, CHU Rennes, Inserm, EHESP, Irset (Institut de recherche en santé, environnement et travail)‐UMR_S 1085 Rennes France; ^10^ INTHERES, Université de Toulouse, INRAE, ENVT Toulouse France; ^11^ Laboratoire de Pharmacocinétique et Toxicologie, CHU de Toulouse Toulouse France; ^12^ Université de Poitiers, CNRS – UMR 7267, CHU de Poitiers, Service de Toxicologie et Pharmacocinétique et INSERM‐CIC 1402 Poitiers France; ^13^ Laboratoire de Pharmacologie et Toxicologie Service de Pharmacologie Médicale, CHU Pellegrin, INSERM U1219 Bordeaux France; ^14^ AP‐HP Nord, Pharmacology Department Bichat Claude‐Bernard University Hospital Paris France; ^15^ Université Paris Cité, INSERM – UMR 1137, IAME Paris France; ^16^ Service de Pharmacologie Clinique, Hôpital Cochin, AP‐HP Université Paris Cité Paris France; ^17^ EA7323, Evaluation des Thérapeutiques et Pharmacologie Périnatale et Pédiatrique Université Paris Cité Paris France

**Keywords:** antiretroviral therapy, cabotegravir, external evaluation, HIV, population pharmacokinetic

## Abstract

Cabotegravir (CAB), combined with rilpivirine, is the first long‐acting injectable therapy approved for HIV‐1 maintenance treatment. While adherence and patient satisfaction have been improved, pharmacokinetic (PK) variability remains a concern. Two population PK models have been developed: one based on data from phase I–III trials and the other on routine clinical data. However, neither model has undergone thorough external evaluation. The aim of this study was to evaluate the predictive performance of these models using an independent prospective dataset to evaluate their suitability to support model‐informed precision dosing (MIPD). External validation was performed using data from the French observational and multicenter (*n* = 14) ANRS0255 CARLAPOP study (736 HIV‐infected patients, representing 2192 concentrations). Models were implemented using MONOLIX software and evaluated using goodness‐of‐fit, prediction‐based, and simulation‐based diagnostics. For Han's model, regarding plasma concentrations following intramuscular administration, Median Prediction Error (MDPE) was −1.2% (PRED) and −4.4% (IPRED); Median Absolute Prediction Error (MDAPE) was 36.6% (PRED) and 17.9% (IPRED). For Thoueille's model, MDPE was −24.2% (PRED) and −9.2% (IPRED); MDAPE was 39.0% (PRED) and 15.3% (IPRED). However, < 70% of predictions were within a 20% error margin with both models. Graphical analyses of Thoueille's model showed systemic bias, particularly in women, nonsmokers, and patients with higher body mass index. Therefore, neither model was considered reliable enough for MIPD application in our population. Although Han et al.'s model demonstrated higher predictive performances, further improvements are required before it can be reliably applied for MIPD in daily routine.

## Study Highlights

- What is the current knowledge on the topic?
  ○ Cabotegravir, in combination with rilpivirine, is the first long‐acting injectable therapy approved for HIV‐1 maintenance. High interindividual variability in plasma concentrations has been described, and several factors associated with virologic failure have been identified. Two population pharmacokinetic (PopPK) models have been developed, demonstrating the effect of various covariates on the pharmacokinetic of cabotegravir.
- What question did this study address?
  ○ This study evaluates the applicability of two cabotegravir PopPK models to model informed precision dosing (MIPD): one developed using clinical trial data (Han et al.) and another based on real‐world therapeutic drug monitoring (TDM) data (Thoueille et al.).
- What does this study add to our knowledge?
  ○ Although Han et al.'s model demonstrated higher predictive performances, further improvements are needed before it can be reliably applied for MIPD.
- How might this change drug discovery, development, and/or therapeutics?
  ○ This highlights the need to develop an adapted model incorporating new, richer real‐world data. This model could be used to describe cabotegravir pharmacokinetic–pharmacodynamic relationships, validate therapeutic concentrations for TDM and support MIPD to improve treatment efficacy.

## 1 Introduction

Cabotegravir and rilpivirine combination (CAB‐RPV) is the first long‐acting (LA) injectable dual therapy approved for the maintenance treatment of HIV‐1 infection. The recommended dosing regimen consists of a loading dose (CAB 600 mg, RPV 900 mg) followed 4 weeks later by maintenance doses of 600/900 mg with a dosing interval of 8 weeks. An optional oral lead‐in period can be proposed before the first injection to evaluate the treatment tolerance [1, 2].

The pharmacokinetics (PK) of LA therapies differ from those of oral formulations, primarily due to flip‐flop kinetics, where absorption becomes the rate‐limiting factor in drug disposition [3, 4, 5, 6]. Phase 3 studies have reported a large interindividual variability and several risk factors for virologic failure have been identified, including suboptimal plasma concentrations during the early stage of the treatment, as recently confirmed in our real‐world cohort [7]. However, consensus on the optimal plasma concentration thresholds required for efficacy has yet to be established [7, 8, 9, 10]. Two population pharmacokinetic (PopPK) models have been developed for CAB: one developed from phase I–III trial data and the other from routine clinical data. These models have highlighted the influence of various covariates on CAB's PK profile. Notably, both identified sex and body mass index (BMI) as significant factors, with women and obese patients showing a lower intramuscular absorption rate constant (ka_LA_) [11, 12]. PopPK models could play a key role in optimizing therapeutic drug monitoring (TDM) of CAB‐RPV. However, their use in clinical practice requires external validation in the same condition of use, which has not yet been formally conducted. Although the model developed by Han et al. has been externally evaluated in four different populations [11, 13, 14, 15], all these evaluations were conducted in clinical trial populations rather than real‐world cohorts and could be enhanced by more quantitative predictive performance metrics.

Moreover, even when models are derived from populations with similar clinical or sociodemographic characteristics, notable differences in PK parameters and variability are frequently observed, potentially leading to inconsistent dosing recommendations. External validation on independent datasets is therefore essential to ensure accuracy, precision, and generalizability in different clinical contexts, making it the most rigorous form of model evaluation [16, 17, 18, 19, 20].

The ANRS‐MIE 0255 CARLAPOP study aims to describe CAB and RPV concentrations and their variability following IM administration in a large cohort of patients under real‐life conditions [7]. In the present analysis, we evaluated the predictive performance of published CAB PopPK models in this population in order to identify a model suitable for model‐informed precision dosing (MIPD) based on TDM. Our work complements the prior publications by providing a rigorous and comprehensive external evaluation of Han's and Thoueille's models, including multiple performance metrics and detailed diagnostic plots in a real‐life setting.

## 2 Materials and Methods

### 2.1 Patient Population

Data from the French multicenter observational study (CARLAPOP study, ANRS0255—PADS 23‐131, *n* = 14 centers) were used to evaluate the models. Ethics approval was granted by the Ethics Committee CPP (Comité de Protection des Personnes, Aix‐Marseille Université, Project ID: 2023‐09‐21‐09) [7].

Baseline demographic, biological, and therapeutic data were collected. All centers followed the recommendation for needle length adjustment based on BMI (50 mm for patients with BMI ≥ 30 kg/m^2^) [21]. Viral load and CD4 count were collected at the different routine follow‐up visits [7]. Qualitative variables are presented as *n* (%), while quantitative variables are expressed as the median [min–max].

### 2.2 Plasma Drug Concentrations

Plasma concentrations were determined at the end of the oral lead‐in phase, 4 weeks after the first injection (M1), 8 weeks after the second (M3), and during follow‐up in accordance with French recommendations [9]. Concentrations collected at M1 ± 3 days or at any other monthly visit (Mx) ±7 days were considered as trough concentrations, whereas all other samples were considered as intermediate‐time concentrations.

CAB plasma concentrations were quantified using a validated multiparametric reverse‐phase ultra‐performance liquid chromatography coupled to tandem mass spectrometry [22]. Among the eight laboratories, six reported a lower limit of quantification (LLOQ) of 10 ng/mL, while the remaining two reported LLOQs of 100 and 200 ng/mL, respectively. All laboratories participated in an external quality assessment program (Asqualab, Paris, France).

### 2.3 Evaluated Models

Two models were identified from the literature; a summary of the studies and model characteristics is provided in Table 1, and the corresponding PK parameter estimates are presented in Table 2. Han et al.'s model was developed using data from phase I, II, and III studies, including both HIV‐1‐infected and healthy adults. The dataset featured rich sampling, with multiple concentration measurements per patient at different time points. A two‐compartment model was used, incorporating distinct first‐order oral and IM absorptions, as well as first‐order elimination. Significant covariates included sex, BMI, split injection, and needle length for ka_LA_, as well as weight and smoking status for clearance. Residual variability was described using a combined error model. The specific distribution of interindividual variability (IIV, *η*) is not explicitly mentioned [11].

**TABLE 1:** Summary of the population pharmacokinetic models for external evaluation.

Reference	Han et al. (2022)	Thoueille et al. (2024)
Population	Infected and uninfected subjects	Infected subjects
Model‐building data set	1647 patients 23,926 samples 16 studies (including LATTE, LATTE‐2, HPTN 077, ECLAIR, FLAIR, and ATLAS)	238 patients 1038 samples (186 oral, 852 IM)
Dosing regimen	Three oral dose levels: 10, 30, and 60 mg Four IM maintenance regimens: 400 mg Q4W, 600 mg Q8W, and 800 mg Q12W	Recommended regimen: 30 mg oral lead‐in, 600 mg Q8W maintenance dosing
Structural model	Two‐compartments Distinct first‐order oral and IM absorptions First‐order elimination Identical volumes and clearances	One‐compartment Distinct first‐order oral and IM absorptions First‐order elimination Identical volume and clearance
Tested covariates	Albumin, total and direct bilirubin, ALP, ALT, AST, GGT, LDH, creatinine, creatinine clearance, urea, UGT1A1 genotype, HIV‐1 viral load and injection‐related factors. Both baseline and time varying	Age, sex, ethnicity, bodyweight, BMI, height, eGFR (CKD‐EPI), and liver cirrhosis (Child‐Pugh score)
Significant covariate on absorption	Sex, BMI, split injection, NDL	Sex, BMI
ka_LA_ formula (1/h)	$0.00073\times \left(1-0.509\;\text{if female}\right)\times \left(1+0.478\;\text{if split}\right)\times {\left(\frac{\mathrm{BMI}}{25.4}\right)}^{-0.766}\times {\left(\frac{\mathrm{NDL}}{1.5}\right)}^{0.478}\times e^{\eta }$	$0.00102\times \left(1-0.405\;\text{if female}\right)\times \left(1-0.999\times \frac{\mathrm{BMI}-25.4}{25.4}\right)\times e^{\eta }$
Significant covariate on clearance	Weight, smoking status	Weight
Clearance formula (L/h)	$\mathrm{CL}:0.151\times {\left(\frac{\mathrm{WT}}{76.6}\right)}^{0.618}\times \left(1+0.174\;\text{if current smoker}\right)\times e^{\eta }$ $Q:0.507\times {\left(\frac{\mathrm{WT}}{76.6}\right)}^{0.618}$	$0.201\times {\left(\frac{\mathrm{WT}}{78}\right)}^{0.460}\times e^{\eta }\times e^{\kappa }$
Significant covariate on volume	Weight	
Volume formula (L)	$\mathrm{Vc}:5.27\times {\left(\frac{\mathrm{WT}}{76.6}\right)}^{0.702}\times e^{\eta }$ $\mathrm{Vp}:2.43\times {\left(\frac{\mathrm{WT}}{76.6}\right)}^{0.702}$	$7.44\times e^{\eta }$
Error model	Combined additive and proportional error	Distinct proportional error for oral and combined (additive and proportional) error for LA
Evaluation method	Basic GOF plots, visual predictive check, bootstrapping (*n* = 500), external validation (647 patients and 5097 concentrations)	Basic GOF plots, visual predictive check, bootstrapping (*n* = 2000), cross‐validation (*n* = 5)
Cabotegravir analytical method	Protein precipitation, followed by HPLC‐MS/MS LLOQ: 10 or 25 ng/mL (later studies)	Multiplex HPLC‐MS/MS LLOQ: 25 ng/mL
Model external evaluations	1—External validation using ATLAS‐2M data 2—Prediction of concentrations in adolescents 3—Prediction of concentrations in women, under real‐world prophylaxis scenarios 4—Prediction of concentrations with an updated tight/gluteal administration model	None

Abbreviations: *η*, interindividual variability; *κ*, inter‐occasion variability; ALAT, alanine aminotransferase; ALP, alkaline phosphatase; ASAT, aspartate aminotransferase; BMI, body mass index (kg/m^2^); CL, central clearance (L/h); eGFR, estimated glomerular filtration rate; GGT, gamma glutamyl transferase; HPLC/MS, high‐performance liquid chromatography coupled to tandem mass spectrometry; IM, intramuscular route; ka, absorption constant rate (h^−1^); LA, long‐acting; LDH, lactate dehydrogenase; LLOQ, lower limit of quantification; NDL, needle length; *Q*, intercompartment clearance (L/h); QM, every month; QW, every week; UGT, UDP‐glucuronosyltransferase; *V*, compartment volume of distribution (L, Vc, central and Vp, peripheral); WT, bodyweight (kg).

**TABLE 2:** Summary of models’ pharmacokinetic parameters.

	Han et al.	Thoueille et al.
Fixed effects		
ka oral (h^−1^)	1.41	1.12
ka IM (h^−1^)	0.000733	0.00102
*F*	75.6%	100%
CL/F (L/h)	0.151	0.201
*Q* (L/h)	0.507	
Vc/F (L)	5.27	7.44
Vp/F (L)	2.43	
Random effects		
IIV (%)		
ka oral	89.4	
ka IM	57.9	37.7
*F*	17.4	
CL/F	23.3	25.6
Vc/F	20.3	107.2
IOV (%)		
CL/F		27.1
Residual error		
Additive (ng/mL)		
Oral	31.9	
IM	31.9	194
Proportional (%)		
Oral	27.3	11.4
IM	27.3	20.4

Abbreviations: CL, central clearance (L/h); F, oral/IM relative bioavailability; IIV, interindividual variability; IM, intramuscular route; IOV, inter‐occasion variability; ka, absorption constant rate (h^−1^); *Q*, intercompartment clearance (L/h); *V*, compartment volume of distribution (L, Vc, central and Vp, peripheral).

Thoueille et al.'s model was developed using real‐life data from people living with HIV (PLHIV). Although the majority of the dataset consisted of sparse data, a subset of 28 patients (11.8%) underwent extensive PK sampling at predefined time points: before the injection and at weeks 1, 2, 4, and 8 post‐dose. A one‐compartment model was used to describe the data, incorporating distinct first‐order oral and IM absorptions, as well as first‐order elimination. Significant covariates included sex and BMI for ka_LA_, and weight for clearance. Residual variability was described using a proportional error model for oral administration and a combined error model for IM administration. Inter‐occasion variability (IOV, *κ*) was also estimated for clearance. Random effects (*η* and *κ*) were assumed to be normally distributed. Interindividual variability was log‐transformed, implying a log‐normal distribution [12]. Notably, more than 70% of the samples in both models were collected following IM administration.

### 2.4 Implementation of the Models

Both models were evaluated using the published parameter estimates, with no re‐estimation or model fitting performed on the CARLAPOP dataset. For population predictions (PRED), model outputs were generated using the fixed typical values of the parameters. For individual predictions (IPRED), empirical Bayes estimates of individual parameters were computed using maximum a posteriori estimation via the MONOLIX software (2024R1), based on each individual's observed concentrations [11, 12, 23].

The CAB concentrations below the LLOQ (BLQ) were treated as left‐censored, non‐negative data (equivalent to the M4 method) [24]. Occasion was set to 1 for the oral phase, then incremented by 1 for each subsequent injection (i.e., 2 for the first injection and so on) within the same subject.

Several methods were tested to impute missing data for covariates identified as significant in the models (weight, height, smoking status). In the absence of statistical differences in the results, the *K*‐Nearest Neighbors method was selected, incorporating all available covariates to ensure robust estimations. This approach was implemented using the *DMwR2* package (version 0.0.2) in R (version 4.3.2), with a number of neighbors set to *k* = 5 [25, 26, 27, 28].

### 2.5 Evaluation of the Predictive Performance of the Models

The predictive performances of the models were evaluated by goodness‐of‐fit (GOF) plots, prediction‐ and simulation‐based diagnostic methods.

#### 2.5.1 Goodness‐of‐Fit

The models were evaluated with standard GOF plots: PRED and IPRED versus the observed CAB concentrations, individual weighted residuals (IWRES) versus time and IPRED.

#### 2.5.2 Prediction‐Based Diagnostics

Percentage error (PE) and absolute percentage error (APE) were calculated for each concentration using the following equations. Where pred_*ij*_ is the *j*th prediction of CAB plasma concentration of the *i*th patient, and obs_*ij*_ is the *j*th actual measurement of CAB plasma concentration of the *i*th patient. Performance metrics were computed separately for PRED and IPRED, then stratified by route of administration. Model accuracy was evaluated using mean and median percentage error (MPE, MDPE), and precision was evaluated using median absolute percentage error (MDAPE) and root mean squared percentage error (RMSPE). In addition, we computed the percentage of PE within ±10%, ±20%, and ±30% (denoted as *F*_10_, *F*_20_, and *F*_30_, respectively) as complementary indicators of prediction acceptability. In our analysis, we applied thresholds of ±20% for bias, < 30% for precision, *F*_10_ > 50%, *F*_20_ > 80%, and *F*_30_ > 90% [16, 20, 29, 30, 31].

(1) $${\mathrm{PE}}_{i,j}=\frac{{\text{pred}}_{i,j}-{\mathrm{obs}}_{i,j}}{{\mathrm{obs}}_{i,j}}$$

(2) $${\mathrm{APE}}_{i,j}=\left|{\mathrm{PE}}_{i,j}\right|$$

(3) $$\mathrm{MPE}=\frac{1}{n}\sum_{i=1}^{n}{\mathrm{PE}}_{i,j}$$

(4) $$\text{MDPE}=\text{median}\left({\mathrm{PE}}_{1,1},\ldots ,{\mathrm{PE}}_{i,j}\right)$$

(5) $$\text{MDAPE}=\text{median}\left(\left|{\mathrm{PE}}_{1,1}\right|,\left|\ldots \right|,\left|{\mathrm{PE}}_{i,j}\right|\right)$$

(6) $$\text{RMSE}=\sqrt{\frac{1}{n}\sum_{i=1}^{n}{\left(\mathrm{pre}d_{i,j}-\mathrm{ob}s_{i,j}\right)}^{2}}$$

(7) $$\text{RMSPE}=\sqrt{\frac{1}{n}\sum_{i=1}^{n}{\left({\mathrm{PE}}_{i,j}\right)}^{2}}$$

(8) $$F_{k}=\frac{1}{n}\sum_{i=1}^{n}\left|{\mathrm{PE}}_{i,j}\right|\times 100\leq k$$

#### 2.5.3 Simulation‐Based Diagnostics

A prediction‐corrected visual predictive check (pcVPC) was performed to explore the agreement between the observations and simulated data. In addition, normalized prediction distribution errors (NPDE) were computed to further evaluate the model's predictive distribution [32, 33].

## 3 Results

### 3.1 Patient Population and Plasma Drug Concentrations

Overall, 736 patients were included, with a median age of 46 years. Of these, 79% were men, 32% were overweight (BMI: [25–30] kg/m^2^) and 11% were obese (BMI ≥ 30 kg/m^2^). The characteristics of the study population are summarized in Table 3. The proportion of missing data was 2.4% for weight, 2.6% for height, and 12% for smoking status. No injections were split. Table S1 presents a comparison between evaluated models and the CARLAPOP study population.

**TABLE 3:** Patients' characteristics at baseline.

Baseline value	*n* (%); median [range]	Missing data, *n* (%)
Sex		
Female	155 (21%)	
Male	581 (79%)	
Age (years)	46 [20–79]	
Time since infection (years)	11 [1–38]	
Bodyweight (kg)	74 [43–130]	18 (2.4%)
BMI (kg/m^2^)	24.5 [16.2–44.8]	26 (3.5%)
< 25	400 (54%)	
[25–30]	232 (32%)	
≥ 30	78 (11%)	
ASAT (IU/L)	24 [11–105]	98 (13%)
ALAT (IU/L)	23 [6–227]	99 (13%)
HIV subtypes		290 (39%)
A	10 (1.4%)	
B	265 (36%)	
Other	171 (23%)	
Plasma HIV RNA (copies/mL)	20 [9–317,000]	20 (2.7%)
< 50	694 (94%)	
[50–200]	15 (2.0%)	
≥ 200	7 (1.0%)	
CD4 count (cells/mm^3^)	763 [144–2067]	284 (38.6%)
≥ 500	372 (50.5%)	
[350–500]	56 (7.6%)	
< 350	24 (3.3%)	
Oral lead‐in	422 (57%)	
Smoking status		90 (12%)
Not current smoker	420 (57%)	
Current smoker	226 (31%)	

Abbreviations: ALAT, alanine aminotransferase; ASAT, aspartate aminotransferase; BMI, body mass index; IU, international unit.

A total of 2192 plasma concentrations (296 after oral administration and 1896 after IM injection) were determined, with a median of 3 [range: 1–18] samples per patient and 1 [range: 1–5] per injection. The observed concentrations versus time are shown in Figure S1. Most IM samples were trough concentrations (84%), a large IIV was observed (ranging from 55% to 72%), as previously described in this cohort [7]. Six IM concentrations were BLQ, and half of them were collected more than 6 months after treatment discontinuation (Figure S4).

### 3.2 External Evaluation

#### 3.2.1 Han Et al.'s Model

**FIGURE 1:**
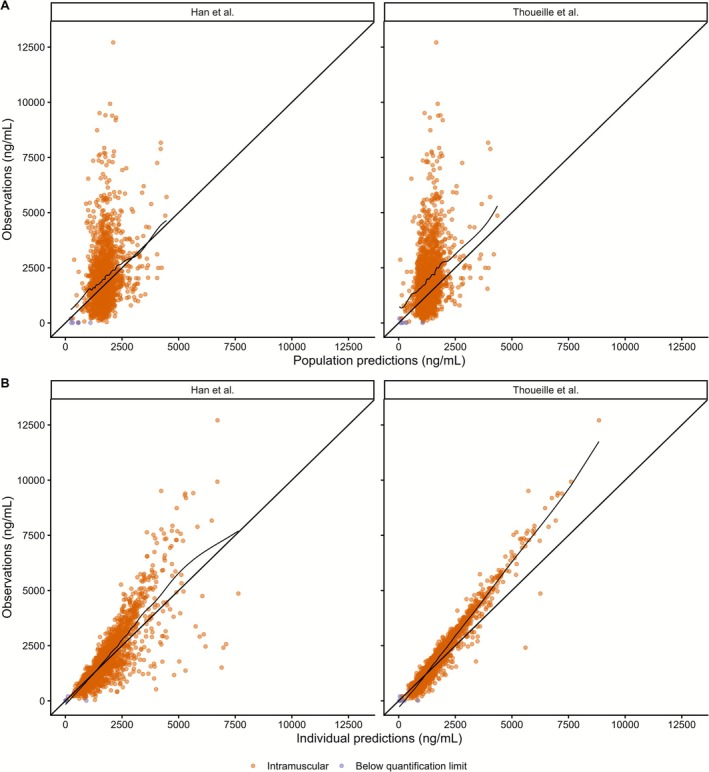
Observed versus predicted concentrations of the models for the intramuscular route. (A) Observations versus population predictions. (B) Observations versus individual predictions.

The GOF plots showed that Han et al.'s model provided predictions of CAB IM concentrations consistent with observations, with the loess smoother closely aligned with the identity line (Figure 1 and Figure S2) and IWRES values symmetrically distributed around zero across IPRED values and time (Figure S3e). The MDPE and MDAPE values were −1.2% and 36.6% for PRED, and −4.4% and 17.9% for IPRED, respectively, showing acceptable accuracy and precision (Table 4). However, the *F*_20_ was only 54.9%, indicating limited reliability of the model predictions at the individual level (Table 4).

**TABLE 4:** Performance of the PopPK models for the intramuscular route: Population and individual predictions.

	MDPE (%)	MDAPE (%)	MPE (%)	RMSPE (%)	RMSE (ng/mL)	*F*_20_ (%)
Population predictions						
Han et al.	−1.2	36.6	43.6	358.4	803	26.7
Thoueille et al.	−24.2	39.0	6.5	276.0	502	24.6
Individual predictions						
Han et al.	−4.4	17.9	17.4	228.4	803	54.9
Thoueille et al.	−9.2	15.3	9.2	210.6	502	68.4

Abbreviations: *F*_20_, proportion of absolute prediction error lower than 20%; MDAPE, median absolute percentage error; MDPE, median percentage error; MPE, mean percentage error; RMSE, root mean squared error; RMSPE, root mean square percentage error.

**FIGURE 2:**
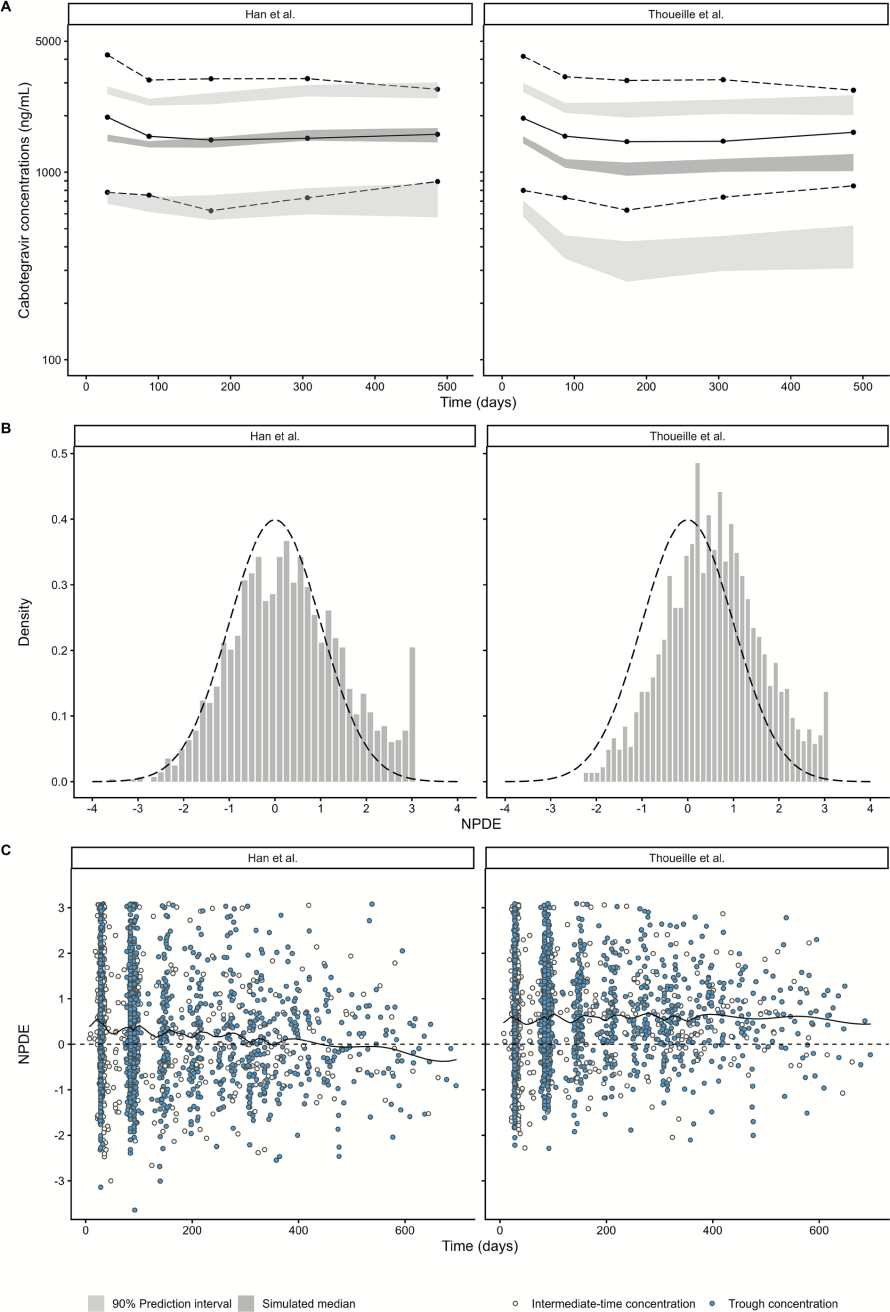
Prediction‐corrected visual predictive checks and normalized prediction distribution errors of the models for the intramuscular route. (A) Prediction‐corrected visual predictive checks. Solid and dashed lines represent the 10th, 50th, and 90th percentiles of the observed data. Shaded areas represent the simulated 90% prediction intervals of the corresponding percentiles. (B) Normalized prediction distribution errors. Histograms are compared with the expected standard normal distribution (black dashed line). (C) Normalized prediction distribution errors versus time. Points represent individual NPDE values, with the loess smoother (solid line) compared to the expected zero line (dashed line). Blue circles represent trough levels, while white circles represent concentrations performed at intermediate times.

**FIGURE 3:**
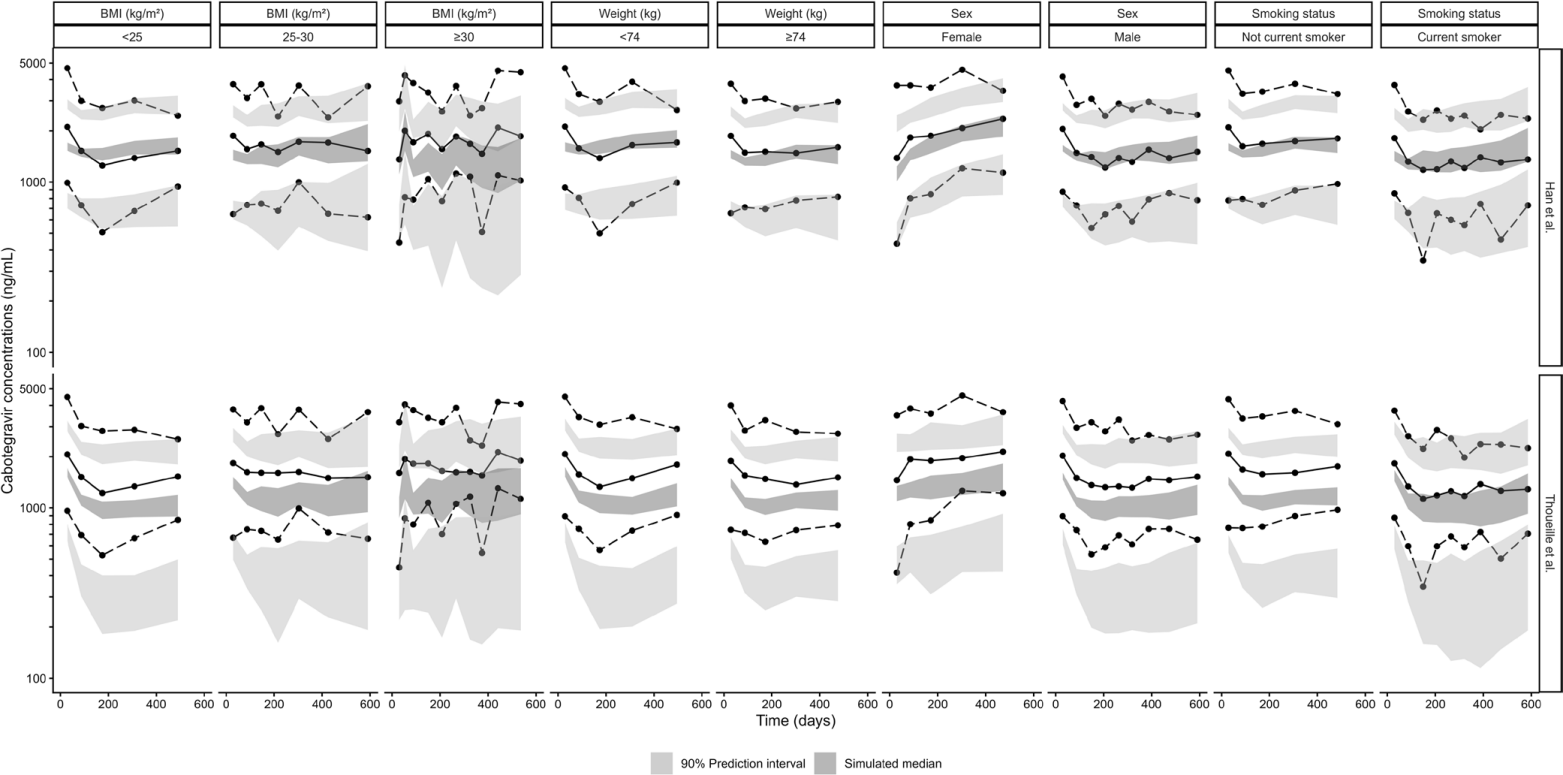
Stratified prediction‐corrected visual predictive checks of the models for the intramuscular route. Solid and dashed lines represent the 10th, 50th, and 90th percentiles of the observed data. Shaded surfaces represent the estimated 90% prediction interval of the 10th, 50th, and 90th percentiles of the simulations. BMI, body mass index.

pcVPC plots (Figures 2A and 3) showed that the model generally captured the overall concentration distribution, although some deviations were observed following the first injections and at higher concentration levels. Stratified VPC (Figure 3) demonstrated similarly satisfactory predictive performance across the different subgroups of weight, BMI, sex, and smoking status. Additionally, for this model, the NPDE distribution was close to the overlaid normal distribution, indicating reasonably accurate model predictions, although some concentrations were underestimated (Figure 2B,C).

#### 3.2.2 Thoueille Et al.'s Model

Bias and precision metrics from Thoueille et al.'s model were within acceptable limits (Table 4): MDPE was −24.2% (PRED) and −9.2% (IPRED); MDAPE was 39.0% (PRED) and 15.3% (IPRED). However, the *F*_20_ (68.4%) remained below the 80% acceptability threshold (Table 4).

The predictions were not consistent with the observations. For PRED, concentrations were generally underestimated (Figure 1A and Figure S2a). For IPRED, the lowest concentrations were overestimated, followed by progressive underestimation as concentrations increased (Figure 1B and Figure S2b). This tendency to underpredict high concentrations was confirmed by a positive bias in the IWRES distribution (Figure S3f) and by pcVPC plots where observed concentrations consistently fell above the prediction intervals (Figures 2A and 3). The NPDE distribution deviated from the theoretical normal distribution (Figure 2B,C). The histogram showed a clear shift toward positive values, suggesting systemic underprediction of concentrations.

Stratified pcVPC analyses showed poorer performance in several subgroups, including patients with higher weight or BMI, women, and nonsmokers (Figure 3). These discrepancies were more pronounced with Thoueille et al.'s model, especially in women and nonsmokers, where predictive accuracy was notably lower.

Results for the oral route are provided in Supporting Information and were overall satisfactory for both models.

## 4 Discussion

Although significant PK variability has been observed with the LA CAB/RPV regimen, its impact on virological outcomes remains uncertain and is likely influenced by multiple factors. In this context, PopPK models represent a valuable tool to predict individual drug concentrations and, ultimately, guide treatment optimization in PLHIV. We evaluated the predictive performance of two published models—one developed from clinical trial data (Han et al.) and the other from routine clinical practice (Thoueille et al.)—in a comparable patient population to assess their suitability for MIPD. To our knowledge, this is the first external evaluation of both models using an independent prospective real‐world dataset. It combines multiple predictive performance metrics with a comprehensive set of diagnostic plots to assess their clinical applicability [11, 12, 13, 14, 15].

Currently, there is no guideline for external evaluation of PopPK models, and clinically acceptable thresholds for predictive metrics remain undefined. Bias metrics (PE, MPE, MDPE) are generally considered acceptable within ±15%–20%, though some studies report thresholds up to ±30%. For precision (MDAPE, RMSPE), values below 30%–35% are commonly used. Additionally, metrics based on the proportions of predictions within fixed percentage thresholds (e.g., *F*_10_, *F*_20_, *F*_30_) are increasingly reported, with values such as *F*_10_ > 35%–40%, *F*_20_ > 50%–60%, or *F*_30_ > 70%–80% often considered indicative of acceptable model performance [16, 20].

Based on MDPE and MDAPE, both models showed acceptable bias and precision in predicting individual CAB IM concentration. However, parametric metrics such as MPE and RMSE were notably high, reflecting their sensitivity to skewed or log‐normal error distributions and outliers. In contrast, nonparametric indicators such as MDPE, MDAPE, and *F*_20_, which rely on medians or counts, are more robust to extreme values. The discrepancy between MPE and MDPE can be attributed to the skewed distribution of PE: while most observations were slightly underpredicted (PE < 0), a few extreme overpredictions (PE ≫ 0) pulled the mean upward, resulting in positive MPE despite negative MDPE. For both models, the largest prediction errors were observed for BLQ concentrations, with PE exceeding 7500%, and PE > 100% was observed in half of the BLQ values (Figure S4). Removing BLQ concentrations led to a substantial reduction in both MPE and RMSPE for Han et al.'s model (from 17% to 12% and 228% to 90%, respectively) and for Thoueille et al.'s model (from 9% to 3% and 210% to 57%, respectively). These results underscored the impact of a small number of extreme values on parametric metrics, and reinforce the importance of using complementary metrics for predictive performance assessment [34].

Overall, Thoueille's model exhibited a systemic bias, characterized by underprediction of concentrations. Although Han et al.'s model adequately described IM concentrations, a tendency to underestimate higher concentrations was observed. Moreover, the low *F*_20_ highlighted the inability of both models to adequately capture a substantial proportion of our observations. Importantly, the fact that IPRED aligned reasonably well with observed data despite poor PRED indicates that much of the predictive performance relied on individual‐specific random effects. In Thoueille's model, this phenomenon was further amplified by the inclusion of an IOV term. This suggests that, although both models can adequately describe concentrations once individual information and variability are incorporated, their ability to provide reliable a priori predictions remains limited. Inaccurate predictions may directly influence clinical decision‐making, potentially leading to inappropriate adjustments in treatment management. Taken together, these findings argue against the suitability of both models for MIPD in our population.

Differences in the studied populations may partly explain the discrepancies between predicted and observed concentrations. Han et al.'s population included both patients and healthy volunteers, with a higher proportion of women (26%), a younger population (median: 36 years) and broader ranges of body weight and BMI ([41.2–168.3] kg and [15.3–69.51] kg/m^2^) compared to our cohort (Table S1).

Han et al.'s model was developed using clinical trial data, including participants from the HPTN077 and ECLAIR studies. While this dataset included a more heterogeneous demographic and greater biological diversity than those included in Thoueille et al.'s model or the present study, it was nonetheless subject to the constraints of clinical trial settings, such as strict inclusion criteria, standardized dosing schedules, and intensive monitoring. These factors can reduce the expression of real‐world PK variability [11, 35, 36]. Han et al. reported an important variability, with an estimated 57.9% IIV on ka, while a subsequent re‐analysis of the HPTN077 data by Yu et al. estimated intraindividual variability at approximately 40%. Such variability is likely to be even greater in real‐world contexts, where treatment adherence, dosing conditions, and clinical practices are more heterogeneous [37, 38].

In contrast, Thoueille et al.'s model, developed using real‐life data from TDM, aligns more closely with our study context. However, with its monocentric design and limited sample size, it may not be able to fully capture the variability observed in our data.

A key difference lies in the structural model: Han et al. used a two‐compartment model, whereas Thoueille et al. relied on a one‐compartment approach. This divergence likely reflects differences in data characteristics. Han et al.'s model was developed using rich sampling schedules, enabling precise characterization of absorption and distribution phases and thus supporting a more complex structure. In contrast, Thoueille et al. developed their model from predominantly sparse real‐world data, which may not adequately capture distribution kinetics, thereby supporting a simpler model structure. The one‐compartment assumption causes a steeper apparent terminal slope compared to a two‐compartment model, as reflected by the 40% higher kaLA of Thoueille's model (0.00102 vs. 0.000733 h^−1^, Table 2).

In addition to these structural differences, the two PopPK models also differ in the covariates identified and their estimated effects on key parameters. Both models retained sex and BMI as significant covariates for ka_LA_, but with important discrepancies. Han et al. predicted a greater decrease in women (−50.9%) compared to Thoueille et al. (−40.5%). Regarding BMI, Han et al. modeled its effect using a power function, resulting in a more gradual decrease in ka_LA_, whereas Thoueille et al. applied a linear relationship. For example, compared to the median BMI (25.4 kg/m^2^), ka_LA_ decreases by 35% in Han's model versus 77% in Thoueille's at a BMI of 45 kg/m^2^. Both models identified weight as a covariate on clearance, resulting in similar clearance estimates, although Han et al. predicted a stronger effect (0.618 vs. 0.460). These discrepancies highlight the models' varying sensitivities to key covariates such as body composition and sex [11, 12].

Han et al.'s model highlighted the impact of injection technique. Indeed, split injections and needle length were identified as significant factors affecting ka_LA_, with an estimated 14.8% increase when switching from a 1.5‐ to a 2‐in. needle. As muscle tissue has greater blood flow than subcutaneous tissue, IM absorption is generally faster. Therefore, proper needle selection is essential to prevent unintended subcutaneous administration, which could delay drug absorption [11, 39, 40]. Han et al. also demonstrated the impact of smoking status, reporting a 17.4% increase in clearance among smokers (Table 1), which can be explained by the fact that smoking is a known inducer of UGT1A1, the primary enzyme involved in CAB metabolism [6, 11, 41].

As discussed by Thoueille et al., the model presents several limitations that may explain the difference in significant covariates compared to Han et al.'s model. The lack of data on smoking status and needle length precluded the evaluation of their impact. This limitation was evident in the stratified VPCs, where Thoueille's model performed poorly in nonsmokers, beyond its overall underprediction of concentrations, whereas Han's model, which accounted for this covariate, provided adequate predictions, underscoring the impact of smoking status. Additionally, the relative bioavailability between oral and IM administration was not retained in Thoueille's model due to the absence of significant differences in their dataset [12]. The estimated IOV on clearance (27.1%) was consistent with previously reported variability in trough concentrations, which ranged from 26% to 52% [7, 42, 43]. However, due to limited data during the absorption phase, IOV could not be estimated for ka_LA_ in Thoueille et al.'s model, even though preliminary analysis had identified its presence [12]. These limitations are likely contributors to the systemic error observed with Thoueille's model. Given the unsatisfactory predictive performance and these differences in covariate effects, a re‐evaluation and refinement of covariate impacts should be considered to achieve a more accurate and reliable representation of CAB PK.

The multicenter design of the CARLAPOP study presents both advantages and challenges: while it captures real‐world variability, it also introduces additional sources of variation. Further investigations could aim to estimate residual variability at the center level to refine model performance.

The inability to identify a single model applicable to all individuals using real‐world data is not surprising and aligns with increasingly reported observations in the literature [20]. Although clinical, biological, and sociodemographic similarities may exist between development and validation cohorts, undetected or undocumented differences often remain. These may result from limited variability in the original datasets or may only become apparent when models are applied to more heterogeneous, real‐life populations. Consequently, rather than seeking a universally applicable model, a more pragmatic approach may involve selecting the most appropriate model for each individual. Depending on patient characteristics and PK profiles, some models may offer better predictive performance than others, supporting a personalized approach to model selection for dose optimization, particularly when multiple concentration measurements are available for a given patient [20, 44].

In conclusion, the model developed by Thoueille et al. demonstrated systemic bias and, although the Han et al. model demonstrated higher predictive performances for the global description of CAB pharmacokinetics, there remains a need to further improve the model for MIPD applications. An adjusted or updated model, incorporating data from published models along with richer real‐world data, such as our cohort, is likely to enhance model transportability [45]. This optimized model could be used to describe the CAB pharmacokinetic–pharmacodynamic relationships and to validate therapeutic concentrations for TDM. In the long term, such a model could be implemented in MIPD tools—including Bayesian estimators or machine learning algorithms—to optimize treatment in PLHIV [46, 47, 48].

## Author Contributions

Quentin Renou, Sihem Benaboud, Alexandre Destere, and Nadège Néant wrote the manuscript; Sihem Benaboud, Alexandre Destere, Nadège Néant, and Caroline Solas designed the research; Sihem Benaboud, Peggy Gandia, Matthieu Grégoire, Jennifer Lagoutte‐Renosi, Minh P. Lê, Sébastien Lalanne, Florian Lemaitre, Patrice Muret, Nadège Néant, François Parant, Gilles Peytavin, Quentin Renou, Caroline Solas, Stéphane Bouchet, and Nicolas Venisse performed the research; Alexandre Destere, Nadège Néant, and Quentin Renou analyzed the data; Peggy Gandia contributed new reagents/analytical tools.

## Funding

This work was supported by Agence Nationale de Recherches sur le Sida et les Hépatites Virales (ANRS)/Maladies Infectieuses Emergentes (Grants ANRS 0255 and ANRS 0691b).

## Conflicts of Interest

The authors declare no conflicts of interest.

## Supporting information


**Figures S1–S4:** psp470180‐sup‐0001‐FiguresS1‐S4.docx.

**Table S1:** psp470180‐sup‐0002‐TableS1.docx.

**Table S2:** psp470180‐sup‐0003‐TableS2.docx.

**Data S1:** psp470180‐sup‐0004‐DataS1.docx.

**Data S2:** psp470180‐sup‐0005‐DataS2.docx.
