# Supplementary material for: External Evaluation of Population Pharmacokinetic Models of Cabotegravir, During Its Oral and Intramuscular Administration in HIV‐Infected Patients
Source: CPT Pharmacometrics Syst Pharmacol. 2026 May 22;15(6):e70180. doi: 10.1002/psp4.70180 (PMC13239761; doi:10.1002/psp4.70180)
Supplement: Supplementary file 1 — Figures S1–S4: psp470180‐sup‐0001‐FiguresS1‐S4.docx. [file PSP4-15-e70180-s004.docx]

**Figure S1 - Cabotegravir concentrations versus time.**


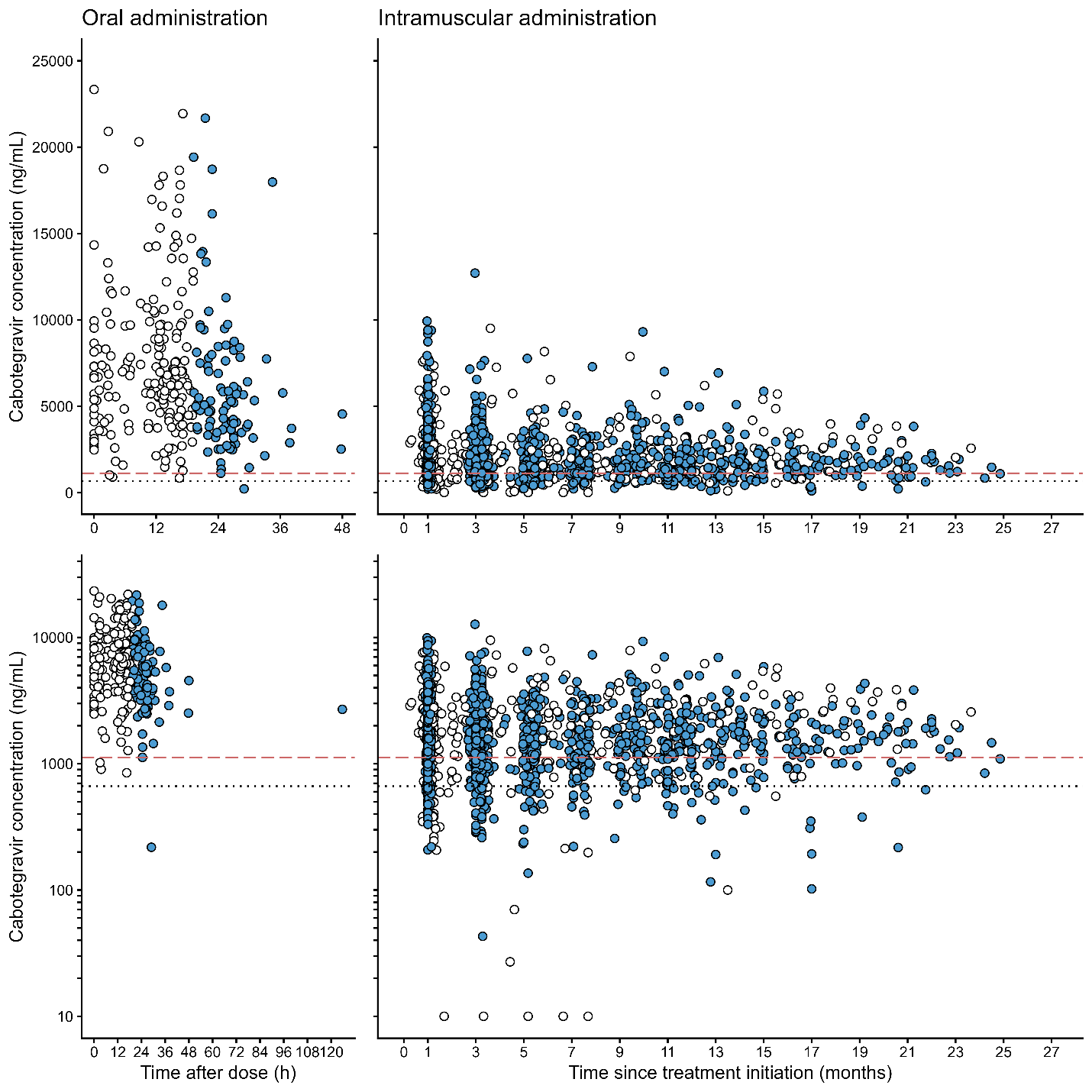


Blue circles represent trough levels, while white circles represent concentrations performed at intermediate times. The black dotted lines correspond to four times the protein-adjusted 90% inhibitory concentration (4 x PA-IC90 - 664 ng/mL), and the red dashed lines represent Q1 trough concentrations (1 120 ng/mL) thresholds reported.^1,2^

**Figure S2 - Observed versus predicted concentrations of the models, for the intramuscular route (log-log scale).**


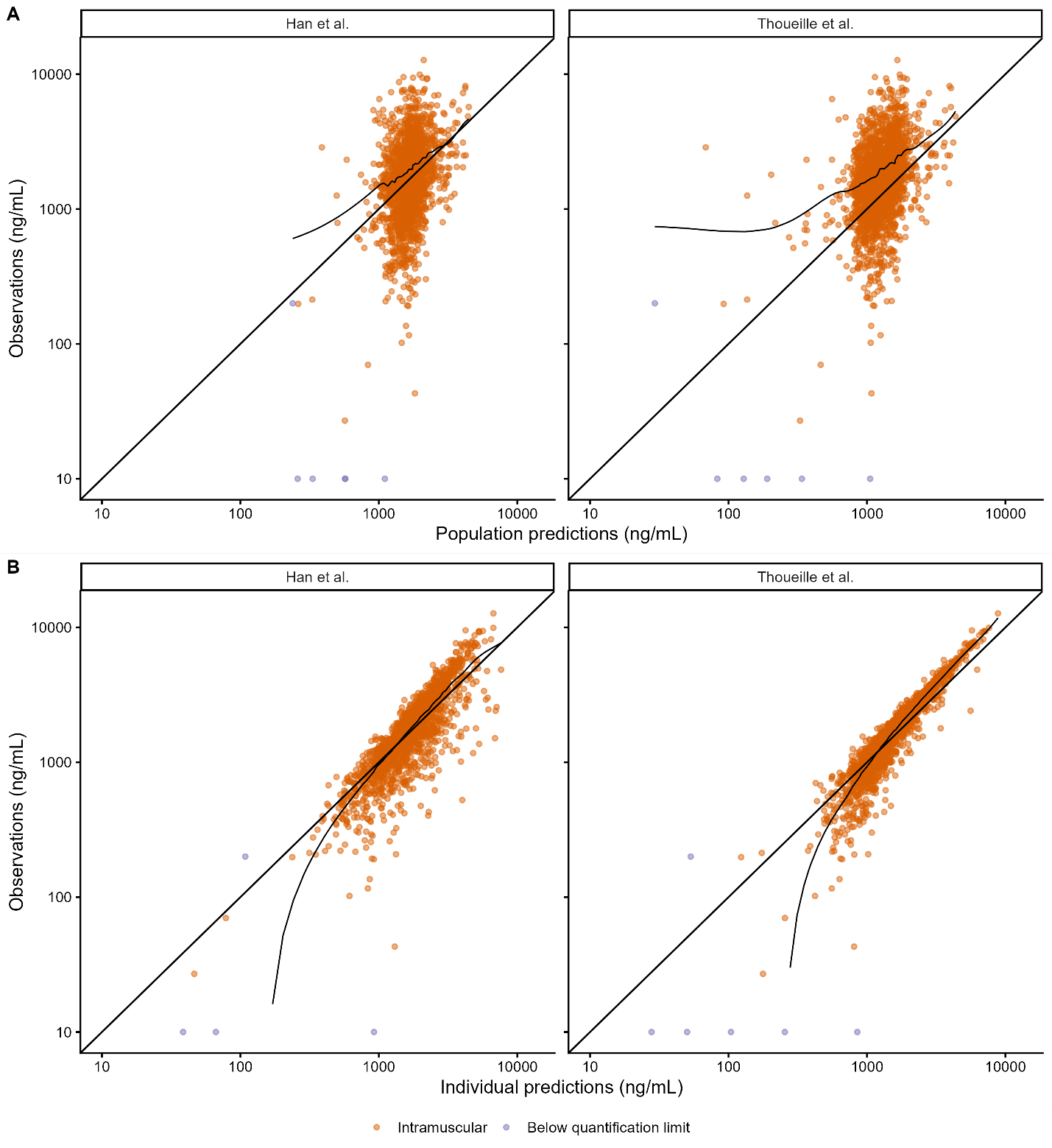


A – Observations versus population predictions. B –Observations versus individual predictions.

**Figure S3 - Goodness-of-fit plots of the models.**


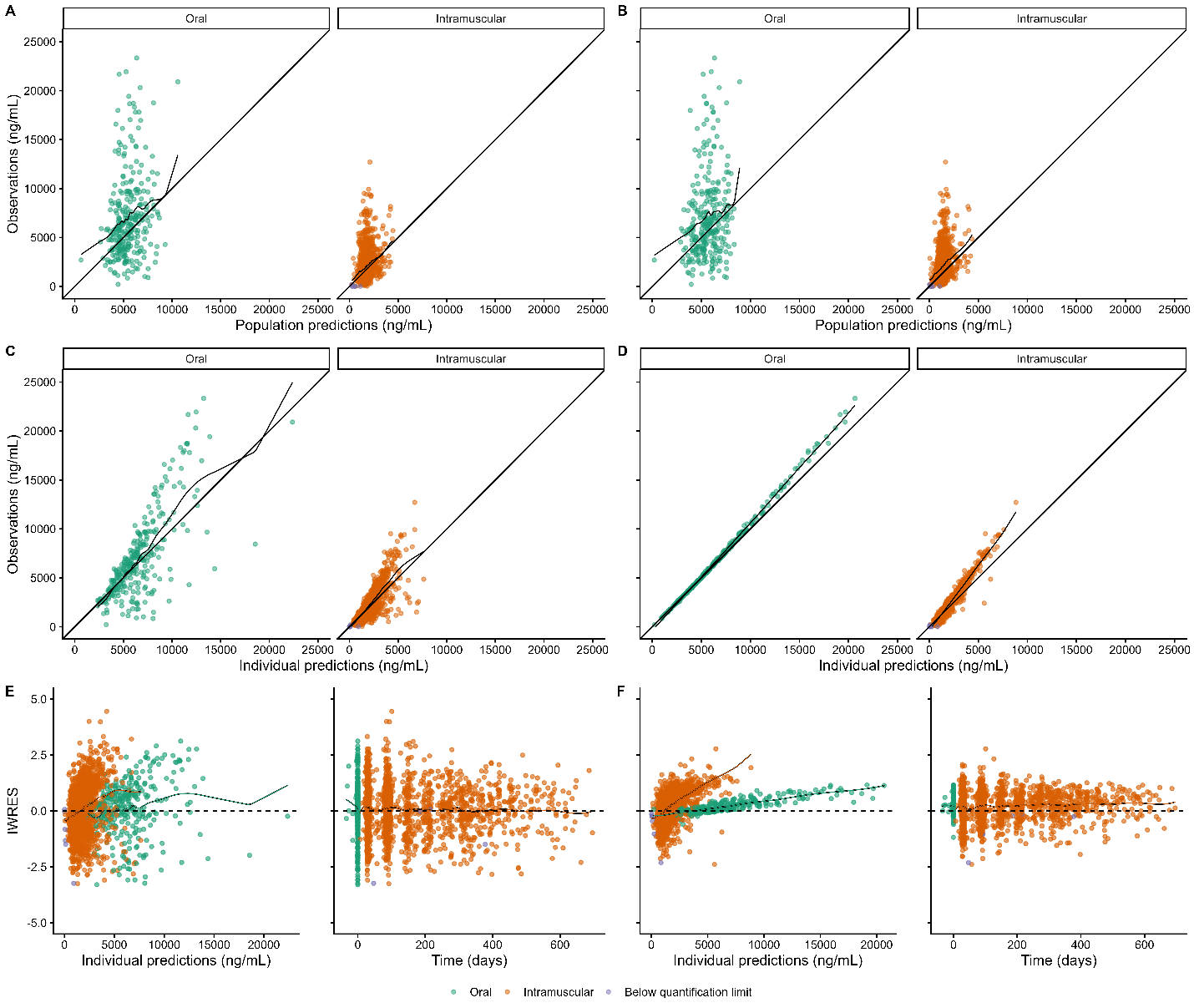


Observations versus population predictions (A, Han et al.’s model.; B, Thoueille et al.’s model), observations versus individual predictions (C, Han et al.’s model.; D, Thoueille et al.’s model), IWRES versus individual predictions and IWRES versus time (E, Han et al.’s model.; F, Thoueille et al.’s model). IWRES, individual weighted residuals.

**Figure S4 - Absolute percentage error of individual prediction for concentrations after intramuscular administration, below the lower limit of quantification or sampled after discontinuation**


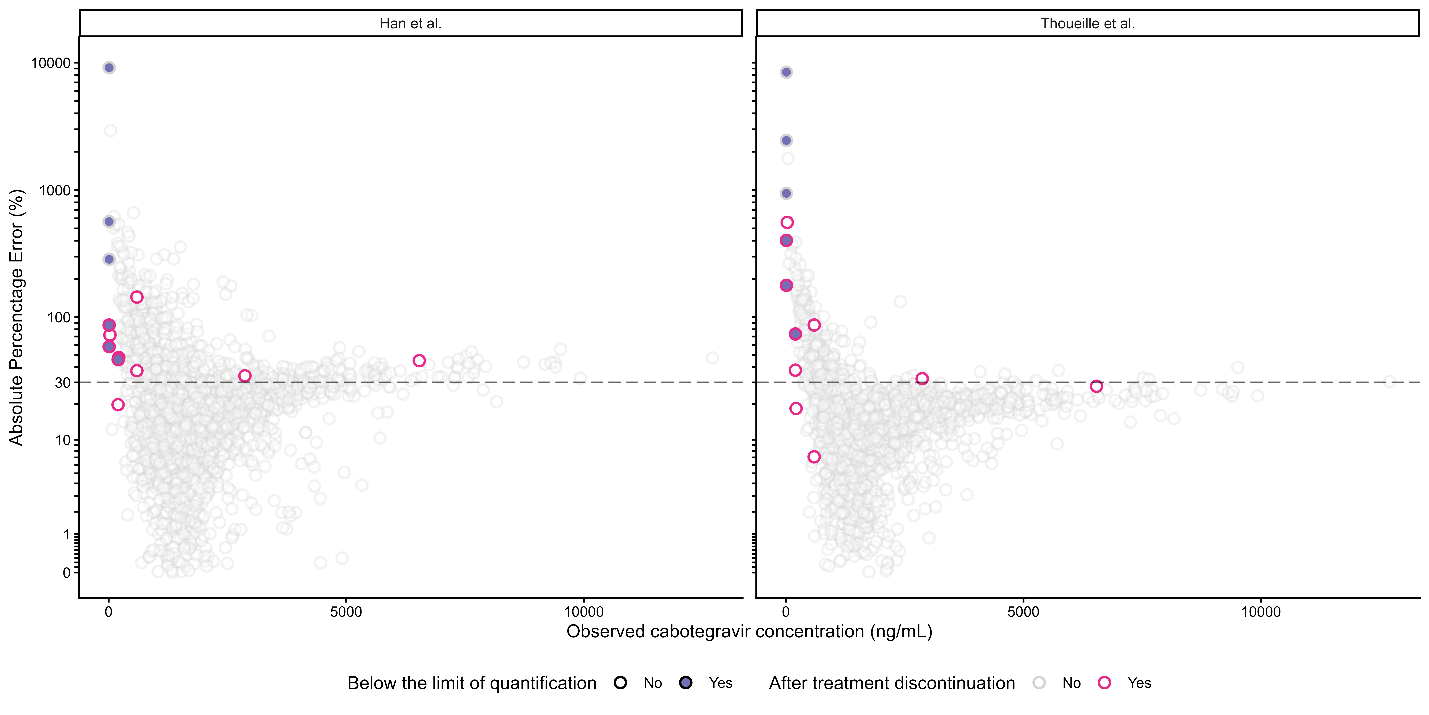


Dashed line represents the reference threshold of 30%.

1. Margolis DA, Brinson CC, Smith GHR, et al. Cabotegravir plus rilpivirine, once a day, after induction with cabotegravir plus nucleoside reverse transcriptase inhibitors in antiretroviral-naive adults with HIV-1 infection (LATTE): a randomised, phase 2b, dose-ranging trial. *The Lancet Infectious Diseases*. 2015;15(10):1145-1155. doi:10.1016/S1473-3099(15)00152-8

2. Cutrell AG, Schapiro JM, Perno CF, et al. Exploring predictors of HIV-1 virologic failure to long-acting cabotegravir and rilpivirine: a multivariable analysis. *AIDS*. 2021;35(9):1333-1342. doi:10.1097/QAD.0000000000002883
