# Supplementary material for: External Evaluation of Population Pharmacokinetic Models of Cabotegravir, During Its Oral and Intramuscular Administration in HIV‐Infected Patients
Source: CPT Pharmacometrics Syst Pharmacol. 2026 May 22;15(6):e70180. doi: 10.1002/psp4.70180 (PMC13239761; doi:10.1002/psp4.70180)
Supplement: Supplementary file 2 — Table S1: psp470180‐sup‐0002‐TableS1.docx. [file PSP4-15-e70180-s003.docx]

**Table S1 – Comparison of baseline characteristics between our cohort and PopPK model’s respective populations.**

| Baseline value | n (%); median [range] | | |
| --- | --- | --- | --- |
| **Study** | ***Han et al.*** | ***Thoueille et al.*** | **CARLAPOP study** |
| **Number of subjects** | 1647 | 238 | 736 |
| **Sex** |  |  |  |
| Female | 424 (26%) | 48 (20%) | 155 (21%) |
| **Age (years)** | 36 [18 - 74] | 46 [20 - 79] | 46 [20 - 79] |
| **Bodyweight (kg)** | 76.6 [41.2 - 168.3] | 78 [50–126)] | 74 [43 - 130] |
| Missing |  |  | 18 (2.4%) |
| **BMI (kg/m²)** | 25.37 [15.3 - 69.51] | 25.4 [18.2 - 43.3] | 24.5 [16.2 - 44.8] |
| <25 |  | 104 (44%) | 400 (54%) |
| [25 - 30[ |  | 103 (43%) | 232 (32%) |
| ≥30 |  | 31 (13%) | 78 (11%) |
| Missing |  |  | 26 (3.5%) |
| **ASAT (IU/L)** | 22 [9 - 352] |  | 24 [11 - 105] |
| Missing |  |  | 98 (13%) |
| **ALAT (IU/L)** | 20 [2 - 153] |  | 23 [6 - 227] |
| Missing |  |  | 99 (13%) |
| **Plasma HIV RNA (copies/mL)** |  |  |  |
| <50 |  | 233 (98%) | 694 (94%) |
| [50 – 200[ |  | 4 (2%) | 15 (2%) |
| ≥200 |  | 1 (<1%) | 7 (1%) |
| Missing |  |  | 20 (3%) |
| **CD4 count (cells/mm³)** |  |  |  |
| ≥500 |  | 186 (78%) | 372 (50%) |
| [350-500[ |  | 23 (10%) | 56 (8%) |
| <350 |  | 29 (12%) | 24 (3%) |
| Missing |  |  | 284 (39%) |
| **Smoking status** |  |  |  |
| Not current smoker | 920 (56%) |  | 420 (57%) |
| Current smoker | 447 (27%) |  | 226 (31%) |
| Missing | 280 (17%) |  | 90 (12%) |

^[[1]](#footnote-1)^

1. ALAT, alanine aminotransferase; ASAT, aspartate aminotransferase; BMI, body mass index; IU, international unit. [↑](#footnote-ref-1)
