# Supplementary material for: External Evaluation of Population Pharmacokinetic Models of Cabotegravir, During Its Oral and Intramuscular Administration in HIV‐Infected Patients
Source: CPT Pharmacometrics Syst Pharmacol. 2026 May 22;15(6):e70180. doi: 10.1002/psp4.70180 (PMC13239761; doi:10.1002/psp4.70180)
Supplement: Supplementary file 3 — Table S2: psp470180‐sup‐0003‐TableS2.docx. [file PSP4-15-e70180-s002.docx]

**Table S2 – Stratified performance of the PopPK models: oral and intramuscular routes, population and individual predictions**

|  | **Route** | **MDPE (%)** | **MDAPE (%)** | **MPE (%)** | **RMSPE (%)** | **RMSE (ng/mL)** | **F10 (%)** | **F20 (%)** | **F30 (%)** |
| --- | --- | --- | --- | --- | --- | --- | --- | --- | --- |
| **Population predictions** | | | | | | | | | |
| ***Han et al.*** |  |  |  |  |  |  |  |  |  |
|  | **Oral** | -10.9 | 32.7 | 16.9 | 146.4 | 2,934 | 16.2 | 28.7 | 45.9 |
|  | **Intramuscular** | -1.2 | 36.6 | 43.6 | 358.4 | 803 | 13.2 | 26.7 | 40.8 |
| ***Thoueille et al.*** |  |  |  |  |  |  |  |  |  |
|  | **Oral** | -9.0 | 35.3 | 19.4 | 138.8 | 551 | 16.2 | 32.4 | 41.6 |
|  | **Intramuscular** | -24.2 | 39.0 | 6.5 | 276.0 | 502 | 12.8 | 24.6 | 38.0 |
| **Individual predictions** | | | | | | | | | |
| ***Han et al.*** |  |  |  |  |  |  |  |  |  |
|  | **Oral** | -6.3 | 18.7 | 23.8 | 125.2 | 2,934 | 26.4 | 52.7 | 69.9 |
|  | **Intramuscular** | -4.4 | 17.9 | 17.4 | 228.4 | 803 | 29.0 | 54.9 | 72.9 |
| ***Thoueille et al.*** |  |  |  |  |  |  |  |  |  |
|  | **Oral** | -1.4 | 1.8 | -2.2 | 4.0 | 551 | 97.3 | 100.0 | 100.0 |
|  | **Intramuscular** | -9.2 | 15.3 | 9.2 | 210.6 | 502 | 30.7 | 68.4 | 86.8 |

^[[1]](#footnote-1)^

1. MDPE, median percentage error; MDAPE, median absolute percentage error; MPE, mean percentage error; RMSPE, root mean square percentage error; RMSE, root mean squared error; F, proportion of absolute prediction error lower than 10, 20 or 30 percents. [↑](#footnote-ref-1)
