# Supplementary material for: External Evaluation of Population Pharmacokinetic Models of Cabotegravir, During Its Oral and Intramuscular Administration in HIV‐Infected Patients
Source: CPT Pharmacometrics Syst Pharmacol. 2026 May 22;15(6):e70180. doi: 10.1002/psp4.70180 (PMC13239761; doi:10.1002/psp4.70180)
Supplement: Supplementary file 4 — Data S1: psp470180‐sup‐0004‐DataS1.docx. [file PSP4-15-e70180-s005.docx]

Members of the CARLA Study Group

**Belfort-Montbéliard:**

Benoît Bailly (Service de Maladie Infectieuses, Hôpital Nord Franche-Comté, Belfort-Montbéliard, France), Vincent Gendrin (Service de Maladie Infectieuses, Hôpital Nord Franche-Comté, Belfort-Montbéliard, France), Timothhee Klopenstein (Service de Maladie Infectieuses, Hôpital Nord Franche-Comté, Belfort-Montbéliard, France), Souheil Zayet (Service de Maladie Infectieuses, Hôpital Nord Franche-Comté, Belfort-Montbéliard, France).

**Besancon:**

Quentin Lepiller (Laboratoire de Virologie, CHU de Besancon, F-25000 Besançon, France, Université de Franche-Comté, CNRS, CHRONO-E (UMR 6249), F-25000 Besançon, France), Fabienne Bozon (Service de Maladies Infectieuses et Tropicales, CHU Besançon, F-25000 Besançon, France), Anne-Sophie Brunel (Service de Maladies Infectieuses et Tropicales, CHU Besançon, F-25000 Besançon, France), Bruno Hoen (Service de Maladies Infectieuses et Tropicales, CHU Besançon, F-25000 Besançon, France), Laurent Hustache-Mathieu (Service de Maladies Infectieuses et Tropicales, CHU Besançon, F-25000 Besançon, France).

**Bordeaux:**

Camille Tumiotto (Virology Laboratory, Pellegrin Hospital, CHU BORDEAUX, Bordeaux, France, University of Bordeaux and CNRS UMR5234, Bordeaux, France), Mojgane Hemssamfar (Services de Médecine Interne et Maladies Infectieuses, Centre Hospitalier Universitaire de Bordeaux (CHU), F-33000, Bordeaux, France, Univ Bordeaux, ISPED, Inserm Bordeaux Population Health, team MORPH3EUS, UMR 1219, CIC-EC 1401, F-33000, Bordeaux, France), Fabrice Bonnet (Services de Médecine Interne et Maladies Infectieuses, Centre Hospitalier Universitaire de Bordeaux (CHU), F-33000, Bordeaux, France, Univ Bordeaux, ISPED, Inserm Bordeaux Population Health, team MORPH3EUS, UMR 1219, CIC-EC 1401, F-33000, Bordeaux, France).

**Dijon:**

Ali Si-Mohammed (Laboratoire de Virologie, CHU de Dijon, Dijon, France), Lionel Piroth (Service des Maladies infectieuses, CHU Dijon, France), Marielle Buisson (Service des Maladies infectieuses, CHU Dijon, France), Sophie Mahy (Service des Maladies infectieuses, CHU Dijon, France), Michel Duong (Service des Maladies infectieuses, CHU Dijon, France).

**Lyon:**

Vinca Icard (Laboratoire de Virologie, Institut des Agents Infectieux, Hôpital de la Croix Rousse, Hospices Civils de Lyon, Lyon, France), Mary-Anne Trabaud (Laboratoire de Virologie, Institut des Agents Infectieux, Hôpital de la Croix Rousse, Hospices Civils de Lyon, Lyon, France), Dulce Alfaiate (Service des maladies infectieuses et tropicales, Hôpital de la Croix Rousse, Hospices Civils de Lyon, Lyon, France), Agathe Becker (Service des maladies infectieuses et tropicales, Hôpital de la Croix Rousse, Hospices Civils de Lyon, Lyon, France), Evelyne Braun (Service des maladies infectieuses et tropicales, Hôpital de la Croix Rousse, Hospices Civils de Lyon, Lyon, France), Florence Brunel (Service des maladies infectieuses et tropicales, Hôpital de la Croix Rousse, Hospices Civils de Lyon, Lyon, France), Matthieu Godinot (Service des maladies infectieuses et tropicales, Hôpital de la Croix Rousse, Hospices Civils de Lyon, Lyon, France), Thomas Perpoint (Service des maladies infectieuses et tropicales, Hôpital de la Croix Rousse, Hospices Civils de Lyon, Lyon, France), Clément Javaux (Service des maladies infectieuses et tropicales, Hôpital de la Croix Rousse, Hospices Civils de Lyon, Lyon, France).

**Marseille:**

Céline Boschi (APHM, Laboratoire de Virologie, Institut Hospitalo-Universitaire Méditerranée Infection, Marseille, France), Philippe Colson (APHM, Laboratoire de Virologie, Institut Hospitalo-Universitaire Méditerranée Infection, Marseille, France), Anne Motte (APHM, Laboratoire de Virologie, Institut Hospitalo-Universitaire Méditerranée Infection, Marseille, France), Véronique Obry-Roguet (TEC, study group) (APHM, Hôpital Sainte-Marguerite, Service d’Immuno-hématologie clinique, Marseille, France), Sylvie Brégigeon (APHM, Hôpital Sainte-Marguerite, Service d’Immuno-hématologie clinique, Marseille, France), Olivia Faucher-Zaegel (APHM, Hôpital Sainte-Marguerite, Service d’Immuno-hématologie clinique, Marseille, France), Hélène Laroche (APHM, Hôpital Sainte-Marguerite, Service d’Immuno-hématologie clinique, Marseille, France), Maeva Dos Santos (APHM, Hôpital Sainte-Marguerite, Service d’Immuno-hématologie clinique, Marseille, France), Isabelle Ravaux (APHM, Institut Hospitalo-Universitaire Méditerranée Infection, Marseille, France), Catherine Dhiver (APHM, Institut Hospitalo-Universitaire Méditerranée Infection, Marseille, France), Christelle Tomei (APHM, Institut Hospitalo-Universitaire Méditerranée Infection, Marseille, France), Amélie Ménard (APHM, Institut Hospitalo-Universitaire Méditerranée Infection, Marseille, France), Matthieu Million (APHM, Institut Hospitalo-Universitaire Méditerranée Infection, Marseille, France), Patrick Philibert (Service d'infectiologie, Hôpital Européèn, Marseille, France), Christina Psomas (Service d'infectiologie, Hôpital Européèn, Marseille, France).

**Mulhouse:**

Jakub Kowalczyk (Service de Dermato-vénéréologie, GHR-Mulhouse Sud Alsace, France), Simona-Loredana Berbescu (Service de Médecine interne, GHR-Mulhouse Sud Alsace, France), Catherine Michel (Service de Dermato-vénéréologie, GHR-Mulhouse Sud Alsace, France).

**Nantes:**

Elisabeth André-Garnier (Nantes Université, CHU Nantes, Laboratoire de virologie, F-44000 Nantes, France), Thomas Drumel (Nantes Université, CHU Nantes, Laboratoire de virologie, F-44000 Nantes, France), Clotilde Allavena (Nantes Université, CHU Nantes, INSERM, Service de Maladies infectieuses et Tropicales, CIC 16 1413, F-44000 Nantes, France), Eric Billaud (Nantes Université, CHU Nantes, INSERM, Service de Maladies infectieuses et Tropicales, CIC 16 1413, F-44000 Nantes, France), Sabelline Bouchez (Nantes Université, CHU Nantes, INSERM, Service de Maladies infectieuses et Tropicales, CIC 16 1413, F-44000 Nantes, France), Cécile Brunet-Cartier (Nantes Université, CHU Nantes, INSERM, Service de Maladies infectieuses et Tropicales, CIC 16 1413, F-44000 Nantes, France), Colin Deschanvres (Nantes Université, CHU Nantes, INSERM, Service de Maladies infectieuses et Tropicales, CIC 16 1413, F-44000 Nantes, France), François Raffi (Nantes Université, CHU Nantes, INSERM, Service de Maladies infectieuses et Tropicales, CIC 16 1413, F-44000 Nantes, France), Véronique Reliquet (Nantes Université, CHU Nantes, INSERM, Service de Maladies infectieuses et Tropicales, CIC 16 1413, F-44000 Nantes, France), Gwennaelle QUERNE (Nantes Université, CHU Nantes, INSERM, Service de Maladies infectieuses et Tropicales, CIC 16 1413, F-44000 Nantes, France), Cecile MEAR-PASSARD (Nantes Université, CHU Nantes, INSERM, Service de Maladies infectieuses et Tropicales, CIC 16 1413, F-44000 Nantes, France), Antoine ASQUIER (Nantes Université, CHU Nantes, INSERM, Service de Maladies infectieuses et Tropicales, CIC 16 1413, F-44000 Nantes, France), Charlotte MOYON (Nantes Université, CHU Nantes, INSERM, Service de Maladies infectieuses et Tropicales, CIC 16 1413, F-44000 Nantes, France), Virginie BESLON (Nantes Université, CHU Nantes, INSERM, Service de Maladies infectieuses et Tropicales, CIC 16 1413, F-44000 Nantes, France), Lio COLLIAS (Nantes Université, CHU Nantes, INSERM, Service de Maladies infectieuses et Tropicales, CIC 16 1413, F-44000 Nantes, France).

**Paris Bichat:**

Anne-Geneviève Marcelin (AP-HP, Sorbonne University, Virology Department, Pitié-Salpêtrière Hospital, Paris, France, Pierre Louis Epidemiology and Public Health Institute, INSERM 1136, Paris, France), Vincent Calvez (AP-HP, Sorbonne University, Virology Department, Pitié-Salpêtrière Hospital, Paris, France, Pierre Louis Epidemiology and Public Health Institute, INSERM 1136, Paris, France), Diane Descamps (AP-HP Nord, Virology Department, Bichat Claude-Bernard University Hospital, Paris, France, Université Paris Cité, INSERM - UMR 1137, IAME, Paris, France), Valentine Ferré (AP-HP Nord, Virology Department, Bichat Claude-Bernard University Hospital, Paris, France, Université Paris Cité, INSERM - UMR 1137, IAME, Paris, France), Yazdanpanah Yazdan (AP-HP Nord, Department of infectious and tropical diseases, Bichat Claude-Bernard University Hospital, Paris, France, Université Paris Cité, INSERM - S 1144, Paris, France), Jade Ghosn (AP-HP Nord, Department of infectious and tropical diseases, Bichat Claude-Bernard University Hospital, Paris, France, Université Paris Cité, INSERM - S 1144, Paris, France), Valérie Pourcher (AP-HP, Sorbonne University, Infectious Diseases Department, Pitié-Salpêtrière Hospital, Paris, France, Pierre Louis Epidemiology and Public Health Institute, INSERM 1136, Paris, France), Marc-Antoine Valantin (AP-HP, Sorbonne University, Infectious Diseases Department, Pitié-Salpêtrière Hospital, Paris, France, Pierre Louis Epidemiology and Public Health Institute, INSERM 1136, Paris, France).

**Poitiers:**

Gwenaël Le Moal (CHU Poitiers, Médecine interne et maladies infectieuses, F-86000 Poitiers, France), David Plainchamp (CHU Poitiers, Médecine interne et maladies infectieuses, F-86000 Poitiers, France).

**Rennes:**

Anne Maillard (Laboratoire de Virologie, CHU de Rennes, Rennes, France), François Benezit (Infectious Diseases and Intensive Care Unit, Pontchaillou University Hospital, Rennes, France), Cédric Arvieux (Infectious Diseases and Intensive Care Unit, Pontchaillou University Hospital, Rennes, France).

**Strasbourg:**

Pierre Gantner (Clinical Virology Laboratory, Strasbourg University Hospital, Strasbourg, France, INSERM UMR_S1109, Strasbourg University, Strasbourg, France), Christine Cheneau (Le Trait d'Union, HIV-infection care center, CHU de Strasbourg, Strasbourg, France), Axel Ursenbach (Le Trait d'Union, HIV-Infection Care Center, Strasbourg University Hospital, Strasbourg, France), Baptiste Hoellinger (Service de Medecine interne, Strasbourg University Hospital, Strasbourg, France), Céline Melounou (Le Trait d'Union, HIV-infection care center, CHU de Strasbourg, Strasbourg, France), David Rey (Le Trait d'Union, HIV-infection care center, CHU de Strasbourg, Strasbourg, France), Yves Hansmann (Service de Medecine interne, Strasbourg University Hospital, Strasbourg, France).
