# Supplementary material for: External Evaluation of Population Pharmacokinetic Models of Cabotegravir, During Its Oral and Intramuscular Administration in HIV‐Infected Patients
Source: CPT Pharmacometrics Syst Pharmacol. 2026 May 22;15(6):e70180. doi: 10.1002/psp4.70180 (PMC13239761; doi:10.1002/psp4.70180)
Supplement: Supplementary file 5 — Data S2: psp470180‐sup‐0005‐DataS2.docx. [file PSP4-15-e70180-s001.docx]

# Monolix files for Han et al. model

Stuctural model

DESCRIPTION:

This PK model has a double extravascular absorption composed of a first-order absorption

(rate constant ka1, fraction F1), and a simultaneous first-order absorption

(rate constant ka2, fraction 1-F1).

The PK model has a central compartment (volume V2), a peripheral compartment

(volume V3, intercompartmental clearance Q), and a linear elimination (clearance Cl).

[LONGITUDINAL]

input = {ka1, ka2, F1, Cl, V2, Q, V3, betaS, REG_SEX, betaT, REG_Tabac}

REG_SEX = {use=regressor}

REG_Tabac = {use=regressor}

EQUATION:

odeType = stiff

; Effect of sex on IM ka

if REG_SEX==1 ; Male

ka2withSEX = ka2

else ; Female

ka2withSEX = ka2 * (1 - betaS)

end

; Effect of Smoke on IM ka

if REG_Tabac==1 ; Current smoker

CLwithTabac = Cl * (1 + betaT)

else ; Non smoker

CLwithTabac = Cl

end

; Parameter transformations

V = V2

k = CLwithTabac/V2

k12 = Q/V2

k21 = Q/V3

PK:

compartment(cmt = 1, volume = V2, concentration = Cc)

absorption(adm = 1, cmt = 1, ka = ka1, p = F1) ; Oral

absorption(adm = 2, cmt = 1, ka = ka2withSEX, p = 1) ; IM

peripheral(k12, k21)

elimination(cmt = 1, k)

OUTPUT:

output = {Cc}

Summary

********************************************************************************

* Model_Han.mlxtran *

* at *

* Monolix version : 2024R1 *

********************************************************************************

ESTIMATION OF THE POPULATION PARAMETERS ________________________________________

Fixed Effects ----------------------------

ka1_pop : 33.8

ka2_pop : 0.0176

beta_ka2_log_NDL : 0.478

F1_pop : 0.756

Cl_pop : 3.62

beta_Cl_log_WT : 0.618

V2_pop : 5.27

beta_V2_log_WT : 0.702

Q_pop : 12.2

beta_Q_log_WT : 0.618

V3_pop : 2.43

beta_V3_log_WT : 0.702

betaS_pop : 0.509

betaT_pop : 0.174

Standard Deviation of the Random Effects -

omega_ka1 : 0.894

omega_ka2 : 0.579

omega_F1 : 0.174

omega_Cl : 0.233

omega_V2 : 0.203

Error Model Parameters -------------------

a : 31.9

b : 0.273

Elapsed time (seconds): 1.2

Exploratory phase iterations: 0 (Fixed number of iterations)

Smoothing phase iterations: 0 (Fixed number of iterations)

_______________________________________________________________________________

ESTIMATION OF THE INDIVIDUAL PARAMETERS ________________________________________

Estimation of the individual parameters by Conditional Distribution ------------

min Q1 median Q3 max shrinkage(%)

ka1 : 26.9 44.5 49.6 54.9 86.9 84

ka2 : 0.00665 0.0158 0.0213 0.028 0.127 22.3

F1 : 0.735 0.751 0.755 0.759 0.782 79

Cl : 1 2.68 3.29 3.83 6.15 -12.3

V2 : 3.6 4.89 5.32 5.72 8.48 82.5

Q : 8.52 11.2 12 12.7 16.9 nan

V3 : 1.62 2.21 2.38 2.55 3.52 nan

betaS : 0.509 0.509 0.509 0.509 0.509 nan

betaT : 0.174 0.174 0.174 0.174 0.174 nan

Elapsed time (seconds): 25

Iterations: 66 (Autostop)

-------------------------------------------------------------------------------

Estimation of the individual parameters by Conditional Mode --------------------

min Q1 median Q3 max shrinkage(%)

ka1 : 17.1 33.6 33.8 33.8 49.1 92.7

ka2 : 0.00504 0.0146 0.0199 0.0287 0.107 10.7

F1 : 0.75 0.756 0.756 0.756 0.78 85.7

Cl : 1.05 2.76 3.33 3.89 6.01 -12.2

V2 : 3.52 4.81 5.2 5.57 7.98 91.2

Q : 8.52 11.2 12 12.7 16.9 nan

V3 : 1.62 2.21 2.38 2.55 3.52 nan

betaS : 0.509 0.509 0.509 0.509 0.509 nan

betaT : 0.174 0.174 0.174 0.174 0.174 nan

Elapsed time (seconds): 1.4

-------------------------------------------------------------------------------

_______________________________________________________________________________

DATASET INFORMATION

Number of individuals: 736

Number of subjects-occasion: 1149

Number of observations (CAB_conc): 2192

Number of doses: 10750

# Monolix files for Thoueille et al. model

Stuctural model

DESCRIPTION:

This PK model has a double extravascular first-order absorption (rate constants ka1 and ka2)

Oral doses must be tagged with ADMINISTRATION ID=1 in the data set and IM doses with ID=2.

The PK model has one compartment (volume V) and a linear elimination (clearance Cl).

[LONGITUDINAL]

input = {F_rel, ka1, ka2, V, Cl, betaS, REG_SEX, betaB, REG_BMI}

REG_SEX = {use=regressor}

REG_BMI = {use=regressor}

EQUATION:

odeType = stiff

; Sex and BMI effect on IM ka

if REG_SEX==1

ka2withSEX_BMI = ka2 * (1 - betaB * ((REG_BMI - 25.2)/25.2)) ; Male

else

ka2withSEX_BMI = ka2 * (1 - betaS) * (1 - betaB * ((REG_BMI - 25.2)/25.2)) ; Female

end

; Elimination

k = Cl / V

PK:

compartment(cmt = 1, amount = Ac, volume = V)

absorption(adm = 1, cmt = 1, ka = ka1, p = F_rel) ; Oral (p = Relative oral/IM bioavailability)

absorption(adm = 2, cmt = 1, ka = ka2withSEX_BMI) ; IM (p = 1, implicit)

elimination(cmt = 1, k = k)

EQUATION:

Cc = Ac / V

Cc_oral = Cc

Cc_IM = Cc

OUTPUT:

output = {Cc_oral, Cc_IM}

Summary

********************************************************************************

* Model_Thoueille.mlxtran *

* at *

* Monolix version : 2024R1 *

********************************************************************************

ESTIMATION OF THE POPULATION PARAMETERS ________________________________________

Fixed Effects ----------------------------

F_rel_pop : 1

ka1_pop : 26.9

ka2_pop : 0.0245

V_pop : 7.44

Cl_pop : 4.82

beta_Cl_log_WT : 0.46

betaS_pop : 0.405

betaB_pop : 0.999

Standard Deviation of the Random Effects -

omega_ka2 : 0.377

omega_V : 1.07

omega_Cl : 0.256

gamma_Cl : 0.271

Error Model Parameters -------------------

b1 : 0.114

a2 : 194

b2 : 0.204

Elapsed time (seconds): 1.9

Exploratory phase iterations: 0 (Fixed number of iterations)

Smoothing phase iterations: 0 (Fixed number of iterations)

_______________________________________________________________________________

ESTIMATION OF THE INDIVIDUAL PARAMETERS ________________________________________

Estimation of the individual parameters by Conditional Distribution ------------

min Q1 median Q3 max shrinkage(%)

F_rel : 1 1 1 1 1 nan

ka1 : 26.9 26.9 26.9 26.9 26.9 nan

ka2 : 0.0125 0.0209 0.0243 0.0276 0.055 42.2

V : 0.958 9.82 12.6 16.7 183 51.9

Cl : 0.722 3.37 4.19 5.09 8.61 16.4

betaS : 0.405 0.405 0.405 0.405 0.405 nan

betaB : 0.999 0.999 0.999 0.999 0.999 nan

Elapsed time (seconds): 25

Iterations: 86 (Autostop)

-------------------------------------------------------------------------------

Estimation of the individual parameters by Conditional Mode --------------------

min Q1 median Q3 max shrinkage(%)

F_rel : 1 1 1 1 1 nan

ka1 : 26.9 26.9 26.9 26.9 26.9 nan

ka2 : 0.0127 0.0205 0.0238 0.0272 0.0518 40.3

V : 1.07 7.15 8 10.7 181 42.3

Cl : 0.777 3.36 4.12 4.93 8.21 21.2

betaS : 0.405 0.405 0.405 0.405 0.405 nan

betaB : 0.999 0.999 0.999 0.999 0.999 nan

Elapsed time (seconds): 6.4

-------------------------------------------------------------------------------

_______________________________________________________________________________

DATASET INFORMATION

Number of individuals: 736

Number of subjects-occasion: 3323

Number of observations (obsid 1): 296

Number of observations (obsid 2): 1896

Number of doses: 5450
